# Antimicrobial Prophylaxis Reduces the Rate of Surgical Site Infection in Upper Gastrointestinal Surgery: A Systematic Review

**DOI:** 10.3390/antibiotics11020230

**Published:** 2022-02-10

**Authors:** Luigi Marano, Ludovico Carbone, Gianmario Edoardo Poto, Natale Calomino, Alessandro Neri, Riccardo Piagnerelli, Andrea Fontani, Luigi Verre, Vinno Savelli, Franco Roviello, Daniele Marrelli

**Affiliations:** Surgical Oncology Unit, Department of Medicine, Surgery and Neurosciences, University of Siena, 53100 Siena, Italy; luigi.marano@unisi.it (L.M.); ludovicocarbone1@gmail.com (L.C.); gianmarioepoto@gmail.com (G.E.P.); natale.calomino@unisi.it (N.C.); alessandro.neri@unisi.it (A.N.); rpiagnerelli@gmail.com (R.P.); fonta79@gmail.com (A.F.); luigi.verre@unisi.it (L.V.); savelli@unisi.it (V.S.); franco.roviello@unisi.it (F.R.)

**Keywords:** surgical site infection, antimicrobial prophylaxis, esophageal surgery, gastric surgery

## Abstract

Surgical site infection occurs with high frequency in gastrointestinal surgery, contributing to the high incidence of morbidity and mortality. The accepted practice worldwide for the prevention of surgical site infection is providing single- or multiple-dose antimicrobial prophylaxis. However, most suitable antibiotic and optimal duration of prophylaxis are still debated. The aim of the systematic review is to assess the efficacy of antimicrobial prophylaxis in controlling surgical site infection rate following esophagogastric surgery. PubMed and Cochrane databases were systematically searched until 31 October 2021, for randomized controlled trials comparing different antimicrobial regimens in prevention surgical site infections. Risk of bias of studies was assessed with standard methods. Overall, eight studies concerning gastric surgery and one study about esophageal surgery met inclusion criteria. No significant differences were detected between single- and multiple-dose antibiotic prophylaxis. Most trials assessed the performance of cephalosporins or inhibitor of bacterial beta-lactamase. Antimicrobial prophylaxis (AMP) is effective in reducing the incidence of surgical site infection. Multiple-dose antimicrobial prophylaxis is not recommended for patients undergoing gastric surgery. Further randomized controlled trials are needed to determine the efficacy and safety of antimicrobial prophylaxis in esophageal cancer patients.

## 1. Introduction

Surgical site infection (SSI) represents a common cause of morbidity and mortality occurring after gastrointestinal surgeries [1], with an average incidence of 10–25% reported in recent literature [2].

Short-term use of antimicrobial prophylaxis (AMP), limited to the intraoperative period or within 24 h postoperatively, prevents postoperative SSI in biliary and colorectal surgery [3,4,5,6,7,8]. Despite recent advances in infection prevention efforts, the efficacy of AMP in esophageal and gastric surgery remains debatable.

Another unsolved topic is the optimal duration of prophylaxis. Few randomized controlled trials (RCTs) as well as prospective cohort studies have investigated the different efficacy between short and extended AMP in upper gastrointestinal surgery.

Nevertheless, there is no worldwide accepted treatment standardization. Recently, the US Centers for Disease Control and Prevention (CDC) and the Healthcare Infection Control Practices Advisory Committee (HICPAC) emphasized the role of short-term AMP in SSI prevention as a measure of quality of care in gastrointestinal surgery [9,10,11]. On the other hand, extended AMP is still adopted in surgical practices, especially in Eastern countries, where extensive lymphadenectomy is routinely performed after surgery for gastric cancer [12].

Short-term prophylaxis seems to be more attractive because it minimizes the outbreak of multidrug-resistant bacteria and *Clostridioides difficile* disease [13], reduces the risk of antibiotic-related adverse events [14,15,16] (i.e., allergic reactions, antibiotics-associated diarrhea [17,18,19,20]), and is cheaper than long-term regimens [21].

Thus, the aim of this systematic review is to investigate the effectiveness of short- compared to long-term AMP in the prevention of SSI in upper-GI surgery.

## 2. Results

### 2.1. Study Selection

**Figure 1:**
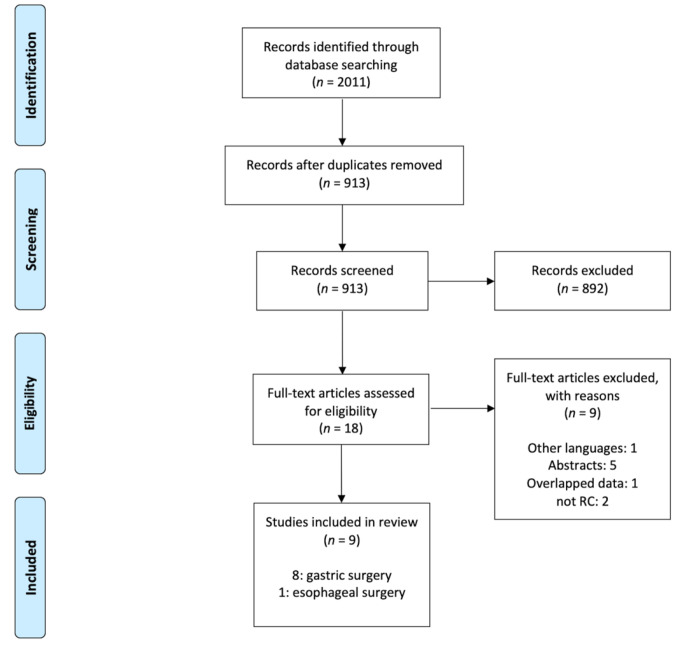
PRISMA flow diagram.

The initial search produced 2011 studies, of which 1088 were excluded because of duplication. The titles and abstracts of the remaining 913 records were screened by PICO criteria and 18 studies fulfilled criteria for eligibility. Nine articles were excluded for the following reasons: one was in Chinese language [22], five were abstract only [23,24,25,26,27], one overlapped data, and two more were prospective observational studies not randomized controlled [28,29] (Figure 1).

After these analyses, a total of nine studies, from 1976 to 2017, were included for the review. Six studies were performed in single centers (USA, Japan, Italy, UK) [30,31,32,33,34,35], while three were multicenter in the Japanese population [36,37,38].

Eight studies included [30,31,32,33,34,36,37,38] patients who underwent gastric surgery, while only one article showed results about esophageal surgery [35].

### 2.2. Reported Outcomes

#### 2.2.1. Antimicrobial Prophylaxis: Do’s or Don’ts

Stone et al. [30], in a cohort of 96 patients that underwent elective gastric surgery, recorded a higher rate of SSI in cases who were untreated (22%) or given antibiotics postoperatively (17%) rather than AMP-treated patients (9%). Similarly, Nichols et al. [31] described the significant efficacy of AMP in SSI control after 39 gastroduodenal procedures, using cephalosporins or placebo (35% vs. 5%, *p* < 0.001) (Table 1).

#### 2.2.2. Types of Antibiotics

Six RCTs investigated the efficacy of cephalosporins, alone [30,31,34,36,38] or combined with nitroimidazole [35], in prophylaxis for gastric and esophageal surgery, respectively, while two studies focused on combined penicillin and inhibitor of bacterial beta-lactamase [36,37]. In addition, Morris et al. [32] highlighted lower incidence of SSI (*p* = 0.03) by using second-generation cephalosporin antibiotics (5%) instead of penicillin (21%) in 78 patients, while Rodolico et al. [33] investigated the SSI rate administering monobactams (0%) and aminoglycosides (27%) in 30 patients (*p* = 0.03) (Table 1).

#### 2.2.3. Antimicrobial Regimen Selection in Gastric Surgery: Single- or Multiple-Dose

Three large RCTs [34,36,38], on patients undergoing open (95.5%) or laparoscopic (4.5%) gastric surgeries, showed that single-dose first-generation cephalosporins were non-inferior to long-term multiple-dose prophylaxis, resulting in equal SSI rate. Interestingly, Mohri et al. reported in their trial the same efficacy in patients treated with combined penicillin and inhibitor of bacterial beta-lactamase [36].

Similar results were described by Takagane et al. in a cohort of 464 patients: the short-term multiple-dose combination of penicillin and inhibitor of bacterial beta-lactamase was as effective as long-term prophylaxis in terms of the risk of occurrence of SSI after total open gastrectomy (8.8% vs. 11%) [37].

Considering the subtypes of SSI (superficial incisional, deep incisional, and organ/space), no differences were reported after the use of different regimens as stated in four RCTs (Table 1).

#### 2.2.4. Antimicrobial Regimen Selection in Esophageal Surgery: Single- or Multiple-Dose

Sharpe et al. [35] conducted their trial in two different populations: malignant esophageal disease (129 patients) and functional esophageal disease (97 patients). In the first group, three different regimens were compared (A: single-dose first-generation cephalosporins and nitroimidazole; B: multiple-dose first-generation cephalosporins; C: multiple-dose first-generation cephalosporins and nitroimidazole), resulting in 9.7%, 7.2%, and 2.2% SSI rate, respectively. If considering B and C regimens as multiple-dose prophylaxis, a non-significant difference arose in SSI rate compared with single-dose (9.4% vs. 9.7%). In the functional disease group, only first-generation cephalosporins were administered, achieving a 6.4% SSI rate with a single-dose and a 4% rate with a multiple-dose regimen. (Table 1)

### 2.3. Quality Assessment

**Figure 2:**
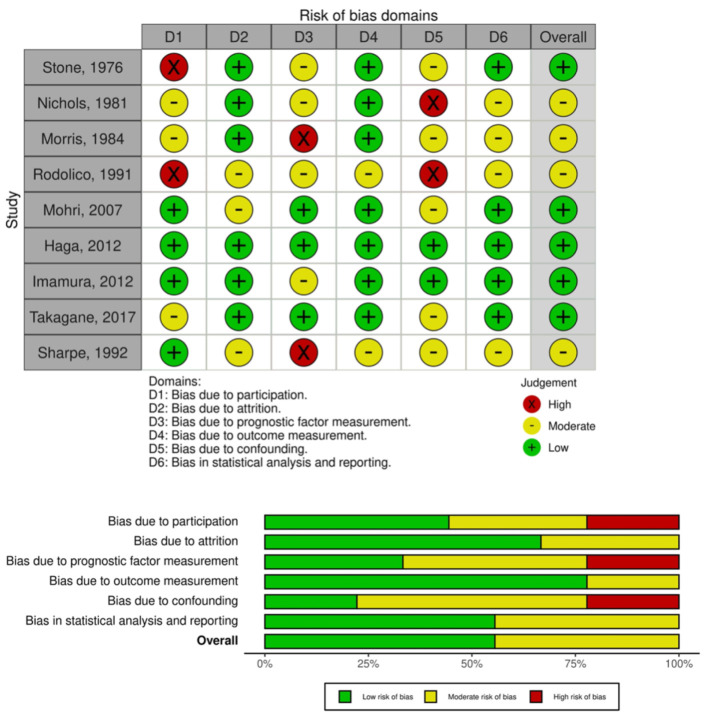
Risk of bias summary: traffic light plot and summary plot.

Among included RCTs, four resulted in moderate risk of bias [31,32,33,35], while five were scored to be of low risk of bias [30,34,36,37,38], according to the Quality In Prognosis Study (QUIPS) tool [39]. Only one trial presented a “high” methodological quality on all items [34] (Figure 2).

## 3. Discussion

Results from our systematic review, based on only randomized studies, clearly show that:

- Antimicrobic prophylaxis reduces SSI in upper-GI surgical patients;
- Preoperative administration of antibiotics results in a more effective reduction in SSI rate;
- Single-dose is non-inferior to multiple-dose prophylaxis in terms of SSIs reduction rate;
- Short-term prophylaxis shows the same effect of long-term prophylaxis in terms of the risk of occurrence of SSI.

Recent literature shows SSI occurring in 10–25% of all abdominal surgical procedures [2,40]. Based on National Nosocomial Infection System (NNIS) reports [41], SSI represents the third most frequently reported nosocomial infection. The high incidence is associated with significant rates of morbidity and mortality, prolonged length of stay, and higher cost amongst hospitalized patients [42,43]. Thus, new AMP schemes, aiming at reducing the risk of infectious complications after gastrointestinal surgery, have been proposed.

Since the 1980s, several authors have studied the role of antibiotics to decrease the risk of infectious complications in esophageal and gastric surgery. Both Stone [30] and Nichols [31] focused on the efficacy of AMP in wound infections rate control compared with no therapy, obtaining similar results to that presented in either biliary or colorectal surgery [8,38,44].

However, the optimal antibiotic class was a matter of debate. Two RCTs, included in this review, described the superiority of cephalosporin and monobactams [32,33], while Takagane et al. conducted their trial administering ampicillin-sulbactam [37]. Nowadays, monobactams are rarely used, probably due to their narrow-spectrum and a lower amount of aerobic Gram-negative bacteria isolated from the abdominal cavity. On the contrary, while first- or second-generation cephalosporins are mainly effective against Gram-positive bacteria, including *S. aureus* and *S. epidermidis*, ampicillin-sulbactam is also effective against anaerobic pathogens. Nevertheless, both drugs showed good efficacy in reducing the incidence of SSI.

Although the effectiveness of appropriate surgical AMP to prevent SSI in indicated procedures was well established, an increasing body of evidence suggested that a single pre-operative dose of antibiotic, with repeated administration intraoperatively when indicated, might be as effective as a prolonged post-operative regimen [45]. In 1999, the US CDC promulgated the first guidelines in gastrointestinal surgery, and recommended to limit intravenous cefazolin antibiotics within the intraoperative period or within 24 h postoperatively [3,46].

However, while prolonged exposure of antibiotics contributes to the emergence of microbial resistance and increased risk of allergic reactions [14,15,16] and kidney injury [47,48,49], a single-dose regimen appears more economical and medically desirable. Moreover, the administration of long-term AMP may result in antibiotics-associated diarrhea, whose incidence varies from 10% to 30% [19,20]. Finally, the reasons behind the longer use of antimicrobial prophylaxis did not seem to be evidence based.

In this regard, three large RCTs [34,36,38], conducted between 2007 and 2012 in Japan, showed no significant difference between intra-operative and long-term AMP, in terms of the overall incidence of SSI following gastric surgery. Similarly, Ohashi et al. led a prospective cohort study, resulting in the same incidences of both incisional and organ/space SSI between single- and multiple-doses in open gastrectomy for gastric cancer [29]. The unambiguous conclusion was that only administration before surgery (including additional intraoperative treatment when surgery exceeds 3 h) is a valid means to reduce the risk of occurrence of SSI.

Three criticisms emerge from these studies: (1) surgical drains may increase the risk of occurrence of SSIs, (2) minimally invasive approach surgery may not expose the wound to bacterial infection, (3) increased hospital readmission, due to occurrence of SSI, may delay the start of adjuvant chemotherapy.

1. An interesting meta-analysis [50] showed that the routine placement of drains was not necessary in elective surgery for gastric cancer, because of higher incidence of postoperative complication and longer hospital stay. Several authors, therefore, encouraged a drain-free strategy [51,52]. On the other hand, Haga et al. encountered no change in incidence of incisional SSI at the drain sites or organ/space SSI around the tips of the drains, advocating for placement of drains at the end of surgery [34].
2. Laparoscopy, in addition to accelerating recovery by decreasing pain and duration of hospital stay, has been shown to be associated with a lower risk of SSI after colorectal surgery [53,54,55]. To our knowledge, few studies compared laparoscopic and open approaches. Minimally invasive surgery was found to be a protective factor for SSI in gastric surgery [56]. In this review, the percentage of patients treated with a laparoscopic approach was too low to draw strong conclusions, and no robotic procedures have been reported yet.
3. The accepted consensus is to perform adjuvant chemotherapy in gastric cancer patients with advanced stages of disease or with pN+ [57]. Nevertheless, a considerable number of patients do not start the therapy or drop out [58]. The minimally invasive approach, early hospital discharge, and no post-operative complications appears to have a great psychological impact and to improve therapeutic compliance [59,60]. On the contrary, SSI occurrence involves long hospitalization, consistent use of antibiotics, and wound drainage or rigorous wound debridement when appropriate [61], resulting in reduced adherence to chemotherapy.

In esophageal surgery, Sharpe’s trial [35] evaluated the efficacy of cefuroxime, due to its broad-spectrum, associated to metronidazole only in cancer patients, to enhance the anaerobic cover provided in more invasive procedures. Despite the use of combined antibiotics and according to recent literature [44,62], the pulmonary infective complication rate was higher in patients that underwent total or partial esophageal resection for malignant disease rather than anti-reflux fundoplication or myotomy. On the other hand, multiple-dose AMP was reported to be effective in preventing SSI, both in cancer and functional disorders of the esophagus. However, most recent studies on a larger cohort of patient encouraged the administration of multidrug single-dose rather than multiple-dose AMP [62,63].

In 2016, WHO conducted a meta-analysis investigating the effect of AMP continuation on SSI risk, and strongly recommended against post-operative continuation of AMP [64,65].

Then, in 2017, the CDC and the HICPAC published an update to their guidelines for prevention of SSI [66,67]. They recommended that a single intravenous dose of preoperative antimicrobial agents should be timed such that the bactericidal concentration is firmly established in the serum and tissues at the time of initial surgical incision [68,69,70,71].

In 2018, the Japan Society for Surgical Infection (JSSI) published the most recent guidelines for the prevention, detection, and management of SSI [44]. Treatment with prophylactic antibiotics was mentioned in gastrointestinal surgery, and, although evidence was limited, administration within 60 min before the surgical incision was preferred. Furthermore, surveillance for more than 30 days after surgery was recommended, including for discharged patients.

Despite this advice, a recent an European multicenter study and a global internet-based point prevalence study found that AMP was still routinely continued for several days after surgery [72]. Otherwise, Kao et al. suggested the SSI rate as reliable measure of hospital quality [10].

This review included RCTs predominantly due to the highest level of evidence they provide with only a few errors. Due to the scarcity of RCTs and other types of studies on this topic, only a limited number of studies were found appropriate to be included for this review. Additionally, although less recent studies cannot be discarded from the selection, data interpretation could be affected by this bias. As most of the studies included in this review were RCTs, the sample size in some of the studies was limited. Moreover, surgeons were not masked to the treatment assignment of each patient and were responsible for identifying any infections occurring after hospital discharge.

## 4. Conclusions

On the basis of our results, we suggest providing AMP during esophagogastric surgery, and to reduce it within the intraoperative period, not exceeding two antimicrobial agents, at the lowest efficacious and safe dose, in order to avoid the emergence of multi-drug resistance. In view of lack of data in literature, further studies are needed to investigate the optimal antimicrobial regimen in esophageal surgery. Additionally, other RCTs should be designed to compare the efficacy AMP between open and minimally invasive approaches.

## 5. Materials and Methods

### 5.1. Searches

A comprehensive and systematic search of the literature was complied with the recommendations of Cochrane Handbook for Systematic Reviews of Intervention [73] and was reported in line with the Preferred Reporting Items for Systematic Review and Meta-analysis (PRISMA) statement [74]. Additionally, the review was submitted to AMSTAR for comprehensive quality score. This study did not require ethical approval and informed consent as all analyses were based on previously published data.

The literature search was conducted in Pubmed and Cochrane databases including all articles published until 31 October 2021. The medical subject headings (MeSH) and keywords “Antimicrobial prophylaxis”, “Antibiotic prophylaxis”, “Gastric surgery”, “Esophageal surgery”, “Esophagogastric surgery”, “Surgical Site Infection”, “Wound Infection”, “Infective complications” were independently undertaken by two investigators (GEP, LC). The keywords were used in all possible combinations to obtain the maximal number of articles.

### 5.2. Selection

The articles were then screened for the presence of the following defined eligibility criteria according to the PICO format [75]: P—population: all patients undergoing esophageal and gastric surgical treatment for any diseases; I and C—intervention and comparator: intravenous antimicrobial prophylaxis; O—outcomes of interest: SSI.

Only published RCTs with full text in English language were included. The reference lists of all selected publications were hand searched for additional relevant articles.

### 5.3. Data Extraction

Two independent reviewers (GEP, LC) screened the retrieved articles from the initial literature search. Two reviewers (GEP, LC) further reviewed independently the eligibility of studies in an abstract form, or if appropriate, in full text, by assessing if the inclusion criteria and outcome measures were met.

Each author decided on trial inclusion using predetermined eligibility criteria:

- RCTs;
Comparison between different doses (no-drug, single-dose if performed during surgery, or multiple-dose if performed also postoperatively) of AMP for esophageal or gastric surgery. Multiple-dose AMP were also distinguished in short-term (within 24 h postoperatively) and long-term (beyond the first postoperative day) regimens;
Studies evaluating SSI, defined by Horan et al. and Mangram et al. [3,46].

Furthermore, the following exclusion criteria were defined:

- Studies lacking full-text available and not published in English language;
RCTs were excluded if antibiotics were administered orally to perform an enteral decontamination [76,77,78].

All detailed reasons for excluding studies were documented. Disagreements were resolved by discussion between the two independent reviewers (GEP, LC); if no agreement could be reached, a third member of the review team was consulted (LM). All identified studies were saved in an Mendeley database.

The following data were recorded: mono- or multi-centricity, country of origin, year of publication, study period, sample size, age of participants, surgical procedure, median or mean duration of follow-up, treatment regimens for estimation of reported outcomes.

We contacted the authors for more detailed data if necessary.

### 5.4. Definition of Surgical Site Infection

Surgical site infection is defined as an infection occurring within 30 days after operation and involving the skin and subcutaneous tissue of the incision (superficial incisional SSI) and/or the deep soft tissue (for example, fascia, muscle) of the incision (deep incisional SSI) and/or any part of the anatomy other than the incision that was opened or manipulated during an operation (organ/space SSI) [15,44,79].

According to the CDC [3,36,46], at least one of the following criteria are required for the diagnosis of SSI:

1. Purulent drainage, with or without laboratory confirmation, from the incision;
2. Microorganism isolated from an aseptically obtained culture of fluid or tissue from the incision;
3. At least one of the following signs or symptoms of infection: pain or tenderness, localized swelling, redness, heat, or fever (>38 °C);
4. Spontaneous wound dehiscence (superficial incisional SSI);
5. Abscess or other evidence of infection involving the fascia, muscle layer or the intra-abdominal cavity found on direct examination, during reoperation, or by histopathological or radiological findings.

### 5.5. Summary Measures

The outcome was to analyze the efficacy and optimal duration of AMP on the rate of SSI after upper-GI surgery.

### 5.6. Quality Assessment

All eligible articles were independently evaluated by two reviewers (GEP, LC) for risk of bias according to Quality In Prognosis Study (QUIPS) tool [39]. Risk of bias was scored as low, moderate, or high for each domain, answering 3–7 prompting questions of the following six items: study participation, study attrition, prognostic factor measurement, outcome measurement, study confounding, and statistical analysis. A final grading of low risk of bias was assigned when three or more of the six items were of “high” methodological quality, while high risk of bias was considered when three or more of the six items resulted to be of “low” methodological quality. Otherwise, a moderate risk of bias was scored. Any reasons for disagreement on certain risk of bias items for a study were discussed and, if no consensus was reached, a third reviewer (LM) was involved in order to obtain a final agreement.

## Figures and Tables

**Table 1:** Basic characteristics of each study included in this systematic review.

Study ID, Year	Country	Study Period	Number of Participants (I Coh/II Coh)	Age (Years)	Surgical Procedure	Duration Follow-Up (Days)	I Cohort	II Cohort	Reported Outcomes
Stone, 1976	USA (single center)	1974–1976	96 (A = 22; B = 27; C = 24; D = 23)	47.6 (2–86)	Open approach Elective admission Gastric surgery	Uncertain	Patients were divided into four treatment categories: A = cefazolin being administered 8–12 h pre-operatively; B = cefazolin being administered just prior to operation; C = cefazolin being administered after operation;	D = not antibiotic.	SSI: Group A = 1 (5%) Group B = 1 (4%) Group C = 4 (17%) Group D = 5 (22%)
Nichols, 1981	USA (single center)	1978–1980	39 (19/20)	I coh: (39–76) II coh: (21–78)	Open approach Gastroduodenal surgery	Uncertain	Patients received a total of 4 g of cefamandole: - 2 g 1 h before operative incision; 1 g 4 and 8 h after incision.	Patients received equal volumes of inert placebo at the same intervals.	SSI (*p <* 0.01): I Cohort = 1 (5%) II Cohort = 7 (35%)
Morris, 1984	USA (single center)	Undefined	78 (40/38)	Undefined	Open approach - Partial gastrectomy for gastric ulcer Total or distal gastrectomy for cancer Vagotomy and pyloroplasty	Uncertain	Patients received cefuroxime 1.5 g after induction of anesthesia.	Patients received mezlocillin 2 g after induction of anesthesia.	Incisional SSI: I Cohort = 1 (2.5%) II Cohort = 5 (13%) Organ/space SSI: I Cohort = 1 (2.5%) II Cohort = 3 (8%) Overall SSI (*p* = 0.03): I Cohort = 2 (5%) II Cohort = 8 (21%)
Rodolico, 1991	Italy (single center)	Undefined	30 (15/15)	I coh: 59 ± 13 (34–84) II coh: 58 ± 12 (32–72)	Open approach - Total or subtotal gastrectomy Gastro-jejunostomy Gastric Raphia	Uncertain	Patients received a total of 3 g of aztreonam: - 1 g 30 min before surgery; 1 g 8 and 16 h after surgery.	Patients received a total of 240 mg of gentamicin: - 80 mg 30 min before surgery 80 mg 8 and 16 h after surgery.	SSI (*p* = 0.03): I Cohort = 0 II Cohort = 4 (27%)
Mohri, 2007	Japan (multicenter)	2001–2004	486 (243/243)	I coh: 68 (22–91) II coh: 68 (23–90)	Open approach Elective admission - Total or distal gastrectomy Gastro-jejunostomy Wedge resection	45	Patients received 1 g of cefazolin or 1.5 g of ampicillin-sulbactam before surgical incision and every 3 h as intraoperative supplements.	Patients received intraoperative schedule and additional doses every 12 h postoperatively, until a total of 7 doses.	Incisional SSI: I Cohort = 14 (5.8%) II Cohort = 11 (4.5%) Organ/Space SSI: I Cohort = 12 (4.9%) II Cohort = 10 (4.1%) Overall SSI: I Cohort = 23 (9.5%) ^1^ II Cohort = 21 (8.6%)
Haga, 2012	Japan (single center)	2007–2010	325 (164/161)	I coh: 68 (33–90) II coh: 68 (39–91)	Open (88.3%) or laparoscopic (11.7%) approach Elective admission Total or distal gastrectomy	30	Patients received 1 g of cefazolin.	Patients received intraoperative schedule and an additional 5 doses every 12 h postoperatively.	Incisional SSI: I Cohort = 14 (8.5%) II Cohort = 7 (4.3%) Organ/space SSI: I Cohort = 11 (6.7%) II Cohort = 6 (3.7%) Overall surgical incision ^2^: I Cohort = 15 (9.1%) II Cohort = 10 (6.2%)
Imamura, 2012	Japan (multicenter)	2005–2007	355 (176/179)	I coh: 66 (36–84) II coh: 65 (35–84)	Open (96%) or laparoscopic (4%) approach Distal gastrectomy	30	Patients received 1 g of cefazolin before surgical incision and every 3 h as intraoperative supplements.	Patients received intraoperative schedule and cefazolin 1 g once after closure and twice daily for two postoperative days.	Superficial incisional SSI: I Cohort = 1 (0.6%) I Cohort = 5 (2.8%) Deep incisional SSI: I Cohort = 0 II Cohort = 0 Organ or space SSI: I Cohort = 7 (4%) II Cohort = 11 (6.1%) Overall SSI: I Cohort = 8 (4.6%) II Cohort = 16 (8.9%)
Takagane, 2017	Japan (multicenter)	2008–2012	464 (228/236)	I coh: 65.5 ± 9.2 II coh: 64.7 ± 10	Open approach Total gastrectomy	30	Patients received 1.5 g ampicillin-sulbactam for 24 h postoperatively.	Patients received 1.5 g ampicillin-sulbactam for 72 h postoperatively.	Superficial SSI: I Cohort = 3 (1.3%) II Cohort = 2 (0.8%) Deep SSI: I Cohort = 0 II Cohort = 2 (0.8%) Organ/space SSI: I Cohort = 17 (7.5%) II Cohort = 23 (9.7%) Overall SSI: I Cohort = 20 (8.8%) II Cohort = 26 (11%) ^3^
Sharpe, 1992	UK (single center)	Undefined	226 (A = 41; B = 42; C = 46; D = 47; E = 50)	63.5 (24–86)	Open approach Esophageal carcinoma (alimentary tract opened): - Esophagectomy Intubation Esophagoplasty Benign disease (alimentary tract not opened): Antireflux Myotomy for achalasia	Uncertain	When alimentary tract was opened, patients were divided into three treatment categories: A = cefoxitina 1.5 g and metronidazole 1 g at induction of anesthesia. When alimentary tract was not opened, patients were divided into two treatment categories: D = treated with cefoxitina 1.5 g on induction of anesthesia.	When alimentary tract was opened: >B = cefoxitina 1.5 g at induction of anesthesia and then cefoxitina 750 mg twice-daily for four days.C = cefoxitina 1.5 g and metronidazole 1 g at induction of anesthesia, then cefoxitina 750 mg twice-daily and metronidazole 500 mg four times per day for four days. When alimentary tract was not opened: E = treated with cefoxitina 1.5 g on induction of anesthesia, then cefoxitina 750 mg twice-daily for two days.	SSI: Group A = 4 (9.7%) Group B = 3 (7.2%) Group C = 1 (2.2%) Group D = 3 (6.4%) Group E = 2 (4%)

^1^ Three patients in the single dose group had both incisional SSI and organ/space SSI. ^2^ Ten patients in the case group and three patients in the control group developed both incisional SSI and organ/space SSI. ^3^ One patient in the control group had both superficial SSI and organ/space SSI.

## Data Availability

The data presented in this study are openly available in Pubmed.

## References

[1] Chodak G.W. (1977). Use of Systemic Antibiotics for Prophylaxis in Surgery. Arch. Surg..

[2] Aga E., Keinan-Boker L., Eithan A., Mais T., Rabinovich A., Nassar F. (2015). Surgical site infections after abdominal surgery: Incidence and risk factors. A prospective cohort study. Infect. Dis..

[3] Mangram A.J., Horan T.C., Pearson M.L., Silver L.C., Jarvis W.R. (1999). The Hospital Infection Control Practices Advisory Committee Guideline for Prevention of Surgical Site Infection, 1999. Infect. Control Hosp. Epidemiol..

[4] Pollock A. (1988). Surgical Prophylaxis—The Emerging Picture. Lancet.

[5] Hall J.C., Watts J.M., Press L., O’Brien P., Turnidge J., McDonald P. (1989). Single-Dose Antibiotic Prophylaxis in Contaminated Abdominal Surgery. Arch. Surg..

[6] Classen D.C., Evans R.S., Pestotnik S.L., Horn S.D., Menlove R.L., Burke J.P. (1992). The Timing of Prophylactic Administration of Antibiotics and the Risk of Surgical-Wound Infection. N. Engl. J. Med..

[7] Polk H.C., Christmas A.B. (2000). Prophylactic antibiotics in surgery and surgical wound infections. Am. Surg..

[8] Bratzler D.W., Houck P.M., Richards C., Steele L., Dellinger E.P., Fry D.E., Wright C., Ma A., Carr K., Red L. (2005). Use of Antimicrobial Prophylaxis for Major Surgery. Arch. Surg..

[9] Hedrick T.L., Sawyer R.G. (2012). The end of postoperative antimicrobial prophylaxis?. Lancet Infect. Dis..

[10] Kao L.S., Ghaferi A.A., Ko C.Y., Dimick J.B. (2011). Reliability of Superficial Surgical Site Infections as a Hospital Quality Measure. J. Am. Coll. Surg..

[11] Jackson S.S., Leekha S., Magder L.S., Pineles L., Anderson D.J., Trick W., Woeltje K.F., Kaye K.S., Lowe T.J., Harris A.D. (2017). Electronically Available Comorbidities Should Be Used in Surgical Site Infection Risk Adjustment. Clin. Infect. Dis..

[12] Japanese Gastric Cancer Association (2011). Japanese gastric cancer treatment guidelines 2010 (ver. 3). Gastric Cancer.

[13] Crabtree T.D., Pelletier S.J., Gleason T.G., Pruett T.L., Sawyer R. (1999). Clinical characteristics and antibiotic utilization in surgical patients with Clostridium difficile-associated diarrhea. Am. Surg..

[14] Childs S.J., Debessonet D.A., Merlin A.S. (1984). Antibiotic prophylaxis in elective genitourinary tract surgery: A comparison of single-dose pre-operative cefotaxime and multiple-dose cefoxitin. J. Antimicrob. Chemother..

[15] Kannan A., Ravichandran M., Sundaramurthi S., Win M., Tara A., Ruo S.W., Sultan W., Yanamala V.L., Mohammed A.R.H., Dominic J.L. (2021). Is Single-Dose Antimicrobial Prophylaxis Sufficient to Control Infections in Gastrointestinal Oncological Surgeries?. Cureus.

[16] Harbarth S., Samore M.H., Lichtenberg D., Carmeli Y. (2000). Prolonged Antibiotic Prophylaxis after Cardiovascular Surgery and Its Effect on Surgical Site Infections and Antimicrobial Resistance. Circulation.

[17] Hogenauer C., Hammer H.F., Krejs G.J., Reisinger E.C. (1998). Mechanisms and Management of Antibiotic-Associated Diarrhea. Clin. Infect. Dis..

[18] Zhang C.-D. (2013). Extended antimicrobial prophylaxis after gastric cancer surgery: A systematic review and meta-analysis. World J. Gastroenterol..

[19] Wiström J., Norrby S.R., Myhre E.B., Eriksson S., Granström G., Lagergren L., Englund G., Nord C.E., Svenungsson B. (2001). Frequency of antibiotic-associated diarrhoea in 2462 antibiotic-treated hospitalized patients: A prospective study. J. Antimicrob. Chemother..

[20] Kreisel D., Savel T.G., Silver A.L., Cunningham J.D. (1995). Surgical Antibiotic Prophylaxis and Clostridium difficile Toxin Positivity. Arch. Surg..

[21] Hall J.C., Mander J., Christiansen K., Reid C., Cooney M., Gibb S.M. (1988). Cost-Efficiency Of A Long-Acting Cephalosporin Agent. ANZ J. Surg..

[22] Li Z., Tong S., Yu B., Tang W., Wu Z., Wang S., Wu Y., Lu W., Luo M., Wang J. (2003). Single-dose ceftriaxone versus multiple-dose cefuroxime for prophylaxis of surgical site infection. Zhonghua Wai Ke Za Zhi.

[23] Sumiyama Y., Kusunoki M. Randomized clinical trial about the period of antimicrobial prophylaxis administration in total gastrectomy for gastric cancer. 2008. JPRN-UMIN000001062.

[24] Svaninger G., Forssell H., Leth R., Lind T., Lundell L., Olbe L. (1987). Antibiotic prophylaxis in high-risk gastric surgery. A prospective, randomized clinical comparison of cefuroxime and doxycycline. Acta Chir. Scand..

[25] Fukushima R., Konishi T., Mohri Y., Noie T., Ono S., Omura K., Sueyoshi S., Takagane A., Kusunoki M., Shibata T. (2014). A prospective randomized study to assess the optimal duration of antimicrobial prophylaxis in total gastrectomy. Surg. Infect..

[26] Han J.H., Jeong O., Ryu S.Y., Jung M.R., Park Y.K. (2014). Efficacy of Single-Dose Antimicrobial Prophylaxis for Preventing Surgical Site Infection in Radical Gastrectomy for Gastric Carcinoma. J. Gastric Cancer.

[27] Aberg C., Thore M. (1991). Single versus triple dose antimicrobial prophylaxis in elective abdominal surgery and the impact on bacterial ecology. J. Hosp. Infect..

[28] Jeong O., Jung M.R., Ryu S.Y., Park Y.-K., Kim M.C., Kim K.H., Ryu S.W., Kwon I.G., Gil Son Y. (2017). Multicenter Phase 2 Study about the Safety of No Antimicrobial Prophylaxis Use in Low-Risk Patients Undergoing Laparoscopic Distal Gastrectomy for Gastric Carcinoma (KSWEET-01 Study). Gastroenterol. Res. Pract..

[29] Ohashi M., Saka M., Katayama H., Okinaka K., Morita S., Fukagawa T., Katai H. (2015). A Prospective Cohort Study To Evaluate the Feasibility of Intraoperative Antimicrobial Prophylaxis in Open Gastrectomy for Gastric Cancer. Surg. Infect..

[30] Stone H.H., Hooper C.A., Kolb L.D., Geheber C.E., Dawkins E.J. (1976). Antibiotic Prophylaxis in Gastric, Biliary and Colonic Surgery. Ann. Surg..

[31] Nichols R.L., Webb W.R., Jones J.W., Smith J.W., LoCicero J. (1982). Efficacy of antibiotic prophylaxis in high risk gastroduodenal operations. Am. J. Surg..

[32] Morris D., Young D., Burdon D., Keighley M. (1984). Prospective randomized trial of single dose cefuroxime against mezlocillin in elective gastric surgery. J. Hosp. Infect..

[33] Rodolico G., Puleo S., Blandino G., Scilletta B., Cavallaro V., Latteri F., Veroux G., Nicoletti G. (1991). Aztreonam Versus Gentamicin for Short-Term Prophylaxis in Biliary and Gastric Surgery. Clin. Infect. Dis..

[34] Haga N., Ishida H., Ishiguro T., Kumamoto K., Ishibashi K., Tsuji Y., Miyazaki T. (2012). A Prospective Randomized Study to Assess the Optimal Duration of Intravenous Antimicrobial Prophylaxis in Elective Gastric Cancer Surgery. Int. Surg..

[35] Sharpe D.A., Renwick P., Mathews K.H., Moghissi K. (1992). Antibiotic prophylaxis in oesophageal surgery. Eur. J. Cardio-Thorac. Surg..

[36] Mohri Y., Tonouchi H., Kobayashi M., Nakai K., Kusunoki M. (2007). Randomized clinical trial of single- versus multiple-dose antimicrobial prophylaxis in gastric cancer surgery. Br. J. Surg..

[37] Takagane A., Mohri Y., Konishi T., Fukushima R., Noie T., Sueyoshi S., Omura K., Ono S., Kusunoki M., Mochizuki H. (2017). Randomized clinical trial of 24 versus 72 h antimicrobial prophylaxis in patients undergoing open total gastrectomy for gastric cancer. Br. J. Surg..

[38] Imamura H., Kurokawa Y., Tsujinaka T., Inoue K., Kimura Y., Iijima S., Shimokawa T., Furukawa H. (2012). Intraoperative versus extended antimicrobial prophylaxis after gastric cancer surgery: A phase 3, open-label, randomised controlled, non-inferiority trial. Lancet Infect. Dis..

[39] Hayden J.A., Côté P., Bombardier C. (2006). Evaluation of the Quality of Prognosis Studies in Systematic Reviews. Ann. Intern. Med..

[40] Laloto T.L., Gemeda D.H., Abdella S.H. (2017). Incidence and predictors of surgical site infection in Ethiopia: Prospective cohort. BMC Infect. Dis..

[41] Centers for Disease Control and Prevention (1996). National Nosocomial Infections Surveillance (NNIS) Report, Data Summary from October 1986–April 1996, Issued May 1996. A Report from the National Nosocomial Infections Surveillance (NNIS) System. Am. J. Infect. Control.

[42] De Lissovoy G., Fraeman K., Hutchins V., Murphy D., Song D., Vaughn B.B. (2009). Surgical site infection: Incidence and impact on hospital utilization and treatment costs. Am. J. Infect. Control.

[43] Badia J.M., Casey A.L., Petrosillo N., Hudson P., Mitchell S., Crosby C. (2017). Impact of surgical site infection on healthcare costs and patient outcomes: A systematic review in six European countries. J. Hosp. Infect..

[44] Ohge H., Mayumi T., Haji S., Kitagawa Y., Kobayashi M., Kobayashi M., Mizuguchi T., Mohri Y., Sakamoto F., Shimizu J. (2020). The Japan Society for Surgical Infection: Guidelines for the prevention, detection, and management of gastroenterological surgical site infection, 2018. Surg. Today.

[45] McDonald M., Grabsch E., Marshall C., Forbes A. (1998). Single-Versus Multiple–Dose Antimicrobial Prophylaxis For Major Surgery: A Systematic Review. ANZ J. Surg..

[46] Horan T.C., Gaynes R.P., Martone W.J., Jarvis W.R., Emori T.G. (1992). CDC Definitions of Nosocomial Surgical Site Infections, 1992: A Modification of CDC Definitions of Surgical Wound Infections. Infect. Control Hosp. Epidemiol..

[47] Morrison L., Zembower T.R. (2020). Antimicrobial Resistance. Gastrointest. Endosc. Clin. North Am..

[48] Blumenthal K.G., Peter J.G., Trubiano J., Phillips E.J. (2018). Antibiotic allergy. Lancet.

[49] Yang X., Zhong H., Xu C., Xu G. (2019). Spotlights on Antibiotic-induced Acute Kidney Injury: The Evidence to Date. Iran. J. Kidney Dis..

[50] Wang Z., Chen J., Su K., Dong Z. (2015). Abdominal drainage versus no drainage post-gastrectomy for gastric cancer. Cochrane Database Syst. Rev..

[51] Liu H.P., Zhang Y.C., Zhang Y.L., Yin L.N., Wang J. (2011). Drain versus No-Drain after Gastrectomy for Patients with Advanced Gastric Cancer: Systematic Review and Meta-Analysis. Dig. Surg..

[52] Shimoike N., Akagawa S., Yagi D., Sakaguchi M., Tokoro Y., Nakao E., Tamura T., Fujii Y., Mochida Y., Umemoto Y. (2019). Laparoscopic gastrectomy with and without prophylactic drains in gastric cancer: A propensity score-matched analysis. World J. Surg. Oncol..

[53] Howard D.P.J., Datta G., Cunnick G., Gatzen C., Huang A. (2010). Surgical site infection rate is lower in laparoscopic than open colorectal surgery. Color. Dis..

[54] Aimaq R., Akopian G., Kaufman H.S. (2011). Surgical Site Infection Rates in Laparoscopic Versus Open Colorectal Surgery. Am. Surg..

[55] Kiran R.P., El-Gazzaz G.H., Vogel J.D., Remzi F.H. (2010). Laparoscopic Approach Significantly Reduces Surgical Site Infections after Colorectal Surgery: Data from National Surgical Quality Improvement Program. J. Am. Coll. Surg..

[56] Inokuchi M., Sugita H., Otsuki S., Sato Y., Nakagawa M., Kojima K. (2015). Laparoscopic distal gastrectomy reduced surgical site infection as compared with open distal gastrectomy for gastric cancer in a meta-analysis of both randomized controlled and case-controlled studies. Int. J. Surg..

[57] De Manzoni G., Marrelli D., Baiocchi G.L., Morgagni P., Saragoni L., Degiuli M., Donini A., Fumagalli U., Mazzei M.A., Pacelli F. (2016). The Italian Research Group for Gastric Cancer (GIRCG) guidelines for gastric cancer staging and treatment: 2015. Gastric Cancer.

[58] Zhao B., Lv W., Lin J. (2020). Delaying adjuvant chemotherapy in advanced gastric cancer patients: Risk factors and its impact on survival outcome. Curr. Probl. Cancer.

[59] Ljungqvist O., Scott M., Fearon K.C. (2017). Enhanced Recovery after Surgery: A Review. JAMA Surg..

[60] Guzman-Pruneda F.A., Husain S., Jones C.D., Beal E., Porter E., Grove M., Moffatt-Bruce S., Schmidt C.R. (2018). Compliance with preoperative care measures reduces surgical site infection after colorectal operation. J. Surg. Oncol..

[61] Mellinghoff S.C., Otto C., Cornely O.A. (2019). Surgical site infections. Curr. Opin. Infect. Dis..

[62] Hochreiter M., Uhling M., Sisic L., Bruckner T., Heininger A., Hohn A., Ott K., Schmidt T., Berger M.M., Richter D.C. (2018). Prolonged antibiotic prophylaxis after thoracoabdominal esophagectomy does not reduce the risk of pneumonia in the first 30 days: A retrospective before-and-after analysis. Infection.

[63] Ruol A., Bertiato G., Boscarino S., Cusinato R., Pascarella M., Tonin E., Santi S., Ancona E. (2000). Short-Term Prophylaxis with Ceftriaxone Plus Metronidazole in Esophageal Cancer Surgery. J. Chemother..

[64] Allegranzi B., Bischoff P., de Jonge S., Kubilay N.Z., Zayed B., Gomes S.M., Abbas M., Atema J.J., Gans S., van Rijen M. (2016). New WHO recommendations on preoperative measures for surgical site infection prevention: An evidence-based global perspective. Lancet Infect. Dis..

[65] Allegranzi B., Zayed B., Bischoff P., Kubilay N.Z., de Jonge S., de Vries F., Gomes S.M., Gans S., Wallert E.D., Wu X. (2016). New WHO recommendations on intraoperative and postoperative measures for surgical site infection prevention: An evidence-based global perspective. Lancet Infect. Dis..

[66] Berríos-Torres S.I., Umscheid C.A., Bratzler D.W., Leas B., Stone E.C., Kelz R.R., Reinke C.E., Morgan S., Solomkin J.S., Mazuski J.E. (2017). Centers for Disease Control and Prevention Guideline for the Prevention of Surgical Site Infection, 2017. JAMA Surg..

[67] O’Hara L.M., Thom K.A., Preas M.A. (2018). Update to the Centers for Disease Control and Prevention and the Healthcare Infection Control Practices Advisory Committee Guideline for the Prevention of Surgical Site Infection (2017): A summary, review, and strategies for implementation. Am. J. Infect. Control.

[68] Bratzler D.W., Dellinger E.P., Olsen K.M., Perl T.M., Auwaerter P.G., Bolon M.K., Fish D.N., Napolitano L.M., Sawyer R.G., Slain D. (2013). Clinical practice guidelines for antimicrobial prophylaxis in surgery. Am. J. Health Pharm..

[69] Steinberg J.P., Braun B.I., Hellinger W.C., Kusek L., Bozikis M.R., Bush A.J., Dellinger E.P., Burke J.P., Simmons B., Kritchevsky S. (2009). Timing of Antimicrobial Prophylaxis and the Risk of Surgical Site Infections. Ann. Surg..

[70] Morita S., Nishisho I., Nomura T., Fukushima Y., Morimoto T., Hiraoka N., Shibata N. (2005). The Significance of the Intraoperative Repeated Dosing of Antimicrobials for Preventing Surgical Wound Infection in Colorectal Surgery. Surg. Today.

[71] Swoboda S.M., Merz C., Kostuik J., Trentler B., Lipsett P.A. (1996). Does Intraoperative Blood Loss Affect Antibiotic Serum and Tissue Concentrations?. Arch. Surg..

[72] Versporten A., Zarb P., Caniaux I., Gros M.-F., Drapier N., Miller M., Jarlier V., Nathwani D., Goossens H., Koraqi A. (2018). Antimicrobial consumption and resistance in adult hospital inpatients in 53 countries: Results of an internet-based global point prevalence survey. Lancet Glob. Health.

[73] Higgins J.P.T., Thomas J. (2021). Cochrane Handbook for Systematic Reviews of Interventions Version 6.2, 2021.

[74] Moher D., Liberati A., Tetzlaff J., Altman D.G., The PRISMA Group (2010). Preferred reporting items for systematic reviews and meta-analyses: The PRISMA statement. Int. J. Surg..

[75] Richardson W.S., Wilson M.C., Nishikawa J., Hayward R.S. (1995). The well-built clinical question: A key to evidence-based decisions. ACP J. Club.

[76] Abis G.S.A., Stockmann H.B.A.C., Van Egmond M., Bonjer H.J., Vandenbroucke-Grauls C.M.J.E., Oosterling S.J. (2013). Selective Decontamination of the Digestive Tract in Gastrointestinal Surgery: Useful in Infection Prevention? A Systematic Review. J. Gastrointest. Surg..

[77] Schardey H.M., Joosten U., Finke U., Staubach K.H., Schauer R., Heiss A., Kooistra A., Rau H.G., Nibler R., Lüdeling S. (1997). The Prevention of Anastomotic Leakage after Total Gastrectomy with Local Decontamination. Ann. Surg..

[78] Farran L., Llop J., Sans M., Kreisler E., Miró M., Galan M., Rafecas A. (2008). Efficacy of enteral decontamination in the prevention of anastomotic dehiscence and pulmonary infection in esophagogastric surgery. Dis. Esophagus.

[79] Gaynes R.P., Culver D.H., Horan T.C., Edwards J.R., Richards C., Tolson J.S., System T.N.N.I.S. (2001). Surgical Site Infection (SSI) Rates in the United States, 1992–1998: The National Nosocomial Infections Surveillance System Basic SSI Risk Index. Clin. Infect. Dis..

